# The Ominous beginning” Perceptions of Smokeless Tobacco Initiation among the Paniya Tribes of Wayanad: A qualitative Study

**DOI:** 10.31557/APJCP.2020.21.6.1615

**Published:** 2020-06

**Authors:** Vineetha Karuveettil, Joe Joseph, Vijay Kumar S, Vinita Sanjeevan, Heljo Joseph Padamadan, Naveen Jacob Varghese

**Affiliations:** *Department of Public Health Dentistry, Amrita School of Dentistry, Amrita Vishwa Vidyapeetham, Kochi, Kerala, India. *

**Keywords:** Tobacco chewing, tobacco habit initiation, ethnography

## Abstract

**Background:**

The prevalence of tobacco chewing, and related oral mucosal lesions is alarmingly high amongst the Paniya tribes of Wayanad. A deeper understanding of their socio-cultural factors, beliefs, attitudes and behaviours would shed greater insights into the indiscriminate use of smokeless tobacco and related products in this community.

**Methods:**

Ethnography was the theoretical framework adopted with network and convenience sampling. Fifteen in-depth interviews and two focus group discussions were conducted among the key informants from within the tribal colonies of Cheepram and Madikkunnu. The data was audio recorded and converted into verbatim transcripts. Thematic content analysis was done using an inductive approach performed using computer-assisted qualitative data analysis software (NVivo).

**Results:**

This study is suggestive of parental influence and peer pressure as the key factors for smokeless tobacco initiation amongst the adolescent. There was a greater predisposition for women to be chewers of tobacco, particularly after marriage. The key factors influencing initiation of the habit amongst men include peer pressure and availability of tobacco at workplace. The role of contextual factors such as enculturation, marginalization and perceived health benefits also play a substantial role in development of this habit.

**Conclusion:**

Targeted strategies for effective tobacco control can be developed through an understanding of the socio-cultural factors leading to initiation of smokeless tobacco use among disadvantaged communities.

## Introduction

The indigenous populations of India are some of the most backward and marginalised communities. The lack of awareness regarding health or risks to health among these communities has resulted in grave concerns regarding their health determinants. Oral health is no exception to this and perhaps more neglected than general health. One of the major risk factors for poor oral health among these tribes is the extensive use of tobacco (Mohindra et al., 2010). 

Wayanad, a hilly district in northern Kerala is home to several tribal communities. Paniya tribes constitutes the largest tribal community in Wayanad and the word ‘Paniya’ translates to ‘Labourer’ or ‘people who does physical work’ (Manojan, 2018). These tribes with a history of enslavement is currently facing extreme marginalization due to lack of social integration and literacy. They are also reported to be less likely to avail scheduled tribe benefits offered by the government compared to other tribes. Their health determinants are poor due to low rates of health care utilization and underreporting of health conditions (Mohindra et al., 2006). 

The prevalence of tobacco usage and related oral mucosal lesions is alarmingly high amongst the Paniya tribes of Wayanad when compared to the general population (Deepa et al., 2013). The use of chewable form of tobacco is very rampant in these communities, particularly among the female population. This is in stark contrast to the rest of the Indian population wherein the use of tobacco by a woman is considered culturally unacceptable. The age of initiation of tobacco use among this population is as early as 16 years (Janakiram et al., 2013). Previous research has indicated that in tribal communities comparable to Paniyas, tobacco initiation may be attributed to familial influence, peer influence, lower perceived smokeless tobacco harm, societal norms and culture (McCleary-Sills et al., 2010; Johnston et al., 2012; Wang et al., 2014; Subramaniyam et al., 2015; Valsan et al., 2016; Chaffe et al., 2019). The implications of tobacco use are many among Paniyas, ranging from health risks to financial burden. Often, addiction is so severe, that a significant proportion of the household income is spent on alcohol and tobacco (Mohindra et al., 2010). 

Any tobacco control program in a community like Paniyas should start with a sound knowledge of the determinants for initiation of this habit. Most of the existent research in this field focuses on estimating prevalence and enumerating risk factors for tobacco chewing. Very few studies have focused on factors leading to the initiation of smokeless tobacco use. Therefore, a qualitative dimension becomes critical to explore how and why a marginalised community has succumbed to tobacco extensively, keeping in mind the cultural and social factors existent in this population. Hence the current study was designed to explore the perceptions of smokeless tobacco initiation among the Paniya tribes of Wayanad.

*Research team and reflexivity*

A team of 6 researchers from the Department of Public Health Dentistry were involved in this study among which, four were post graduate residents and two were postgraduate degree holders in Public Health Dentistry (MDS). The team has been regularly involved with the tribal colonies as a part of various outreach activities and as a result has a good rapport with the population. 

**Table 1:** Demographic Characteristics of Tribes Interviewed

Characteristics	n	Mean
Age (years)	25	38±16.49
Gender		
Male	5	
Female	20	
Tobacco chewing		
Chewer	15	
Ex chewer	4	
Non – Chewer	6	
Age of Onset of tobacco chewing	19	14±3.66
Educational Status		
Not educated	4	
Lower Primary	4	
Upper primary	6	
Higher Secondary	11	

## Materials and Methods

*Study Design*

Ethnography was the theoretical framework adopted in this study to explore the influence of socio-cultural factors associated with tobacco chewing. Network and convenience sampling were employed. The duration of the study was for four months until the data saturation was achieved. In-depth interviews and Focus Group Discussions based on a semi-structured guide was conducted to explore key factors leading to smokeless tobacco initiation. The study cleared institutional ethics committee approval. Written informed consent were obtained from all the participants, as the tribes could put in their own signatures.

*Study Setting and Sample Population*

The study setting comprised of two tribal colonies of Wayanad, Kerala (Cheepram and Madikkunnu), which are predominated by the Paniyas. A total of 15 participants (four males and 11 females) above 18 years of age participated in the in-depth interviews. This included tobacco chewers, previous chewers and non- chewers. A social worker and a health worker, maintaining a close relationship with the tribes were involved in guiding the team through the colonies. They were also interviewed to gain a deeper insight. Out of 20, five refused to consent for in-depth interviews, stating they were uncomfortable in discussing their tobacco use. 

*Study procedure and Analysis*

A semi structured interview guide was prepared based on available literature and the interviews were conducted in regional language (Malayalam). The participants were directly approached by researchers and face-to face interviews were conducted. The in-depth interviews were conducted in secluded places and the participants had total privacy. The Focus Group Discussions were conducted in the community halls of the two colonies, areas familiar and comfortable to the tribes. Each FGD had five participants. Literate participants read the consent form themselves, while the interviewer read it out to those who were illiterate. 

Each in-depth interview lasted around 45 minutes and focus group discussions were conducted for one hour. The interview proceedings were recorded by a note keeper and both the in-depth interviews and FDGs were audio recorded with consent. The recordings were converted to verbatim transcripts at the end of the day and emerging new guides or themes were added for the subsequent interview next day. 

After attaining data saturation, thematic content analysis was performed by coding, categorizing and ultimately developing themes from the transcribed data. Each transcript was reviewed by two researchers (VK and VS) and emerging themes were developed involving a third author in case of disagreement (JJ). Consensus on codes, categories and themes were made by discussions conducted regularly. The data was organised and managed by a computer assisted qualitative research software NVivo (Version 11).

In order to ensure rigor in research design, data was collected from various sources involving two data collection sites, negative cases and participants with previous history of chewing tobacco in order to ensure transferability. Member checking for the study results was conducted among tribes and the feedbacks were recorded to warrant credibility. Triangulation was performed by a third author in case of disagreement on coding and theme development to ensure dependability.

## Results

The characteristics of the tribal participants are described in Table 1. The age of the participants ranged from 18 to 68 years. The mean age of onset of tobacco chewing is 14±3.66 years among the participants. Majority of the participants were females as most men were reluctant to discuss their smokeless tobacco use. 

Results were interpreted starting with the habit history, material availability of the tobacco products and the tribe’s existent health beliefs, in order to get a background for evaluating the tobacco initiation. This was continued by themes specific to tobacco initiation which included enculturation, parental influence, marriage, peer pressure, workplace availability, perceived health benefits and marginalization. Negative cases among the tribes, who refrained from using tobacco products were also interviewed to challenge generalizations.

*Background for tobacco initiation*

*Habit History, Frequency and Gender Disparities*

Tobacco was introduced to India in the early 1,600s by the Portuguese traders. Since then chewing tobacco with betel quid (paan) was a socially accepted practice and was a part of culture and religious customs in India. With globalization this habit was considered socially unacceptable and it started disappearing from the communities (Gupta et al., 2016). Still people who are deeply rooted in their culture continue this habit and Paniya tribes of Wayanad are one among them. Tobacco chewing is widespread among these tribes, with users ranging from children, pregnant women to the elderly. This is in contrast with other substance abuse case studies. Even though both the sexes chew tobacco there exists a predisposition for women to chew more as men has shifted to other forms of tobacco. 

“When we chew, tobacco leaves are a must, along with crushed arecanut and lime. These are kept in the betel leaves and are rolled to form the betel quid. That is how we chew tobacco.” Tribal Female 3

“Most of the tribes chew tobacco around 2 to 3 times daily, but there are few who chew more than 10 times a day. The habit of chewing is present in both the sexes. But there exists a greater indulgence of this habit among women. The common reasons being, boredom and men having shifted to other forms of tobacco, like beedis and cigarettes.” Health worker male

*Addiction and occasions*

The addiction towards chewing is deeply rooted in the tribes. Even food is considered as a secondary necessity over tobacco chewing. It is essential, as per their religious customs to keep tobacco products in social gatherings and celebrations, e.g. marriage, festivals and funerals. Women consume tobacco even during pregnancy. It is only when they visit temples that they refrain from chewing tobacco. 

“I once advised a young girl to stop chewing. I warned her she might get cancer. Her reply was that she can starve for weeks, but she can’t stop chewing tobacco” FGD Tribal male 4.

“Yes, most of our social gatherings like marriage, engagement and even funeral, will have tobacco, as it is a part of our culture. It must be offered to our guests” Tribal Female 3.

*Health beliefs*

With the emergence of media and improved health awareness the tribes are aware of the harmful effects of tobacco (Janakiram et al., 2016). The health beliefs of tribes in relation to tobacco chewing are twofold, there are people who believe it is harmful to their health but still continue using it and there are sections which do not believe it is harmful even after repeated warnings from the health workers. The reason for disbelief is because they rarely see the harmful side of tobacco and even if they do, they do not correlate it to tobacco chewing. 

“I don’t think they consider it serious enough to stop it. In spite of what we say they have been using it for generations, and they will say [my parents used to chew, my grandparents used to chew, they had no problems then, so why should we stop chewing?]” Health Worker Male

“I am trying to quit this habit because I am taking medicines for other ailments and doctors have advised me to stop chewing while taking them. This has made me aware of the harmful effects of tobacco” Tribal Female. 

“Yes, I have seen advertisements which say that it is injurious to health. I partly believe it and partly don’t because I have heard people who do not consume tobacco are also getting cancer in the mouth. So that confuses me” Tribal Female 8.

*Material availability*

The supply and demand of tobacco products are high among the Paniya Communities. This has led to a flourishing tobacco industry and tobacco is easily available. Till date there is no cultivation of tobacco in the region and the products are imported from nearby states. For a shop to thrive in the colonies, they must keep tobacco products. The tribes usually frequent such shops and buy other things too, thereby adding to the profit. Selling tobacco to minors is prohibited in India, but this is violated in this community. 

“There are no exclusive shops that sells tobacco products, it is available in all shops. If tobacco is not available in a shop, the tribes stop frequenting these shops. If the shopkeepers keep betel nut and tobacco in their shops, people will shop more frequently. So even if they increase 1 to 2 rupees for other things, we don’t really mind.” FGD Tribal Female 14.

“One person (A non-tribe seller) has told me that “Selling tobacco and chewing products is more profitable than selling gold”. They make a lot of money by selling these products. People will go to their shops and buy other things too, if it’s available there. So, this might be tempting them to frequent that shop.” Health Worker Male

*Perceived factors contributing to the initiation of chewing tobacco*

*Theme 1: Enculturation*

There are many factors leading to tobacco initiation among this community, which are directly or indirectly influencing them to succumb to this habit. Majority of these factors can be traced back to their culture. Tobacco chewing is a part of Paniya culture and is being practiced across generations. This is an integral part of their cultural gatherings and festivals. The habit is so deeply rooted that there is a sense of identity attached to tobacco chewing. 

“I think most of the tribes have evolved with the exception of Paniyas, probably because they are still stuck to their culture and their roots. They have not integrated into the general society. That is the general opinion about Paniyas.” Health Worker Male

“It started as a part of our culture. In Hindu religious function we keep betel nut with tobacco. We still use it in our marriage functions. Chewing tobacco is a part of our lifestyle. There is also a custom to give tobacco to the children.” FGD 1 Tribal female 12.

*Theme 2: Parental Influence*

The tribes live in colonies which is a collection of individual houses belonging to each family. Their social bonding and familial relationships stand strong. The grandparents and parents look after their children till they are of marriageable age and until then they all share a close bond. Tobacco chewing starts very young in Paniyas, even among children as young as 10 years of age. This initiation comes from the family members and is in line with the social learning theory of Bandura, where a habit is initiated based on observational learning and modelling. The child sees the mother or grandparents chewing and they are tempted to try this (Akers and Lee, 1996). Most of the parents belonging to the current generation do not give their children tobacco, but they do use it themselves. So, by watching their elders chew, the habit gets rooted in children.

“Ever since, I saw my mother using this, I wanted to try it. I did try it when I was younger and now it has gradually developed into a habit.” FGD Tribal female 13

“I have a daughter, when she sees me chewing, she always asks me to give her betel quid. But we don’t want to encourage this habit, so we don’t give her.” Tribal female 13.

“The parents cannot ask their children to stop chewing because they themselves chew. Only if they lead by example will the child stop taking up this habit.” Health Worker Male.

Some parents encourage their children to chew tobacco contrary to the majority. They provide children with money to buy the tobacco products for themselves and as a token of gratitude encourage them to start using tobacco. This is also used as a technique in managing children. To calm a crying child or a child throwing tantrums, the parents give children tobacco to chew. So, the next time the child cries he or she will demand tobacco to stop crying and the habit is initiated like this.

“When children cry inconsolably, it is common practice for the parents to give the child tobacco. That’s how the habit usually start” Tribal Female 9.

*Theme 3: Marriage*

The tribal women get married very young, as early as 12 years of age. Majority of the tribal women starts chewing after marriage. One of the reasons for this is that, young girls are restricted from chewing by their parents. After getting married they are introduced into this habit by their mother in laws or husbands leading to habit formation. There is also a symbol of adulthood attached to getting married, with the notion that women can take decisions for themselves, leading to them leaning to taking up this habit. Majority of the women are housewives and after marriage they don’t continue education or work. This leads to boredom and chewing tobacco turns into a hobby. 

“Most parents won’t allow their children to chew in their house. But once they get married, especially women, they start the habit of chewing. Their husbands might buy them or they themselves will buy tobacco and chew.” Tribal Female 7.

“I would say women indulge in this habit a bit more to overcome boredom. It is usually after marriage that women will start chewing.” Tribal Male 3.

*Theme 4: Peer Pressure*

The tribes socialize well among themselves; they form peer groups and work groups. When they see people of similar age groups chewing, the habit is taken up by some of them. Sometimes tobacco chewing is more of a group activity than individual. In all most all their group meetings and gatherings, tobacco is available abundantly. The peer pressure to chew is noticeably more among young tribal men. 

“People use this frequently, because they see other people using it and they want to try it, that’s how this becomes a habit. When there are group meetings or get-togethers, we get access to this product abundantly.” Tribal Female 5.

“People get together often in the community and start chewing. Some of them may force others to start this habit. By initiating others into this habit, they are in fact harming them.” Tribal Female 7.

*Theme 5: Workplace availability*

Tobacco products are abundantly available in workplaces of the tribal men. Most men are labourers involved in strenuous jobs in the cultivation fields or other areas. Because of the physical demand of their work, they buy tobacco, or their employer landlords provide them. It is done under the pretext of getting enhanced energy to perform such tasks. Another reason stated being tobacco kills hunger pangs and thereby the workers can skip having a meal. Thus, they can work longer to get paid extra or to finish off the work. 

“Majority of our men are contract workers. To avoid one meal, they chew tobacco, so they won’t feel hungry. They also claim that they do feel more energetic to perform such strenuous tasks after chewing.” FGD Tribal Female 12.

“The usual trend among Paniyas is to study till 3rd or 4th grade and then the boys will drop out of school to start working. At the workplace, they get tobacco in abundance.” Social Worker Male.

*Theme 6: Marginalization*

The Paniya tribes usually keep to themselves and don’t socialize much to the outside world. Very few of the Paniya men travel outside for work. They still follow their culture strongly and due to a lack of integration with the outside society, they do not understand that tobacco chewing is considered aversive among other communities. The impact of marginalization prevents them from understanding that this habit is not socially acceptable. This leads to social normalization of tobacco chewing.

“Other tribes from Wayanad like Kurichiya, Kurimar are relatively well-educated and are employed as teachers, doctors, engineers etc. They have a better lifestyle and they stay away from these habits. This was possible due to education and social integration with general population. The Paniyas and Uralis are the most backward among tribes in terms of social development. They are still a marginalized section due to lack of education and social isolation.” Social Worker Male.

*Theme 7: Perceived health benefits*

Tribes consider chewing tobacco to be beneficial to their health. Majority of them start chewing because there is an existent belief, that chewing tobacco relieves toothache and prevents development of dental caries. It is common for parents to give tobacco to their wards to relieve toothache. The tribes also use tobacco as a mouth freshener and are accustomed to the flavour of tobacco. There also exists a tendency to use tobacco products as a measure to control hunger pangs. 

“I started chewing tobacco to relieve tooth ache which gradually progressed into a habit. None of my children chew and they advise me to stop. But I usually tell them that I am chewing to relieve my toothache.” Tribal Female 10.

“When I have Kanji (Indian rice soup), I have a feeling of bad breath, in order to control that I use tobacco after meals.” Tribal Female 2.

“One of the reasons why Paniyas take up this habit is that, it has helped them to tolerate their hunger. When they fast for a long time, they don’t feel the hunger pangs if they are chewing.” Health Worker Male.

“We have learned from our elders that, when there is a swelling of gums or tooth ache, applying tobacco over that area can relieve it. The pain will usually subside after a while”. Tribal Female 3. 

*Negative Cases*

Apart from majority of tobacco chewers, there exists a small proportion of tribes who refrain from this habit or have stopped chewing. This can be attributed to an increase in awareness related to harmful effects of tobacco use from the media, social workers and health workers. Another reason is the strong flavour of tobacco which certain people couldn’t endure. Some have reported nausea and drowsiness when they tried tobacco for the first time, which made them to not continue this habit. Socialization has also played an important role in this, as other tribes like Kurumar, Kurichiya who are more integrated to the society stay away from this habit and this can be attributed to social reasons rather than health reasons. Some of them shared that since this habit is addictive and there are chances of not being able to stop this habit once started, they hold back the urge to chew.

“I know that it can cause cancer. I have seen advertisements on TV and newspapers that warn the use of tobacco.” Tribal Female 7.

“Tobacco chewing is relatively less among tribes like Kurumar and Kuruchiya because they have integrated more into general society. You don’t find these tribes chewing. They may be smoking, they may be drinking, but they don’t chew. They have adapted to the societal norms compared to Paniyas.” Health Worker Male.

“Usually people who stay in the tribal colonies have this habit. My family is living far away from the colonies so none of us including my husband and children have developed this habit.” Tribal Female 5.

“Once when I had toothache one of my relatives suggested a remedy, whereby tobacco is placed in the affected area. I tried that but felt nauseous and drowsy due to which I stopped using that for tooth ache. You can also get cracks or burns on your lips and your teeth get stained so it’s not good to chew. I know that those who take up this habit has tough time to quit this.” Tribal Female 5.

## Discussion

This study was devised to explore the perceptions of tobacco initiation in the tribal population of Wayanad using qualitative methods. The study confirms that Paniyas start chewing tobacco as early as 14 years of age, which emphasizes the adolescent onset of tobacco initiation. This is in line with various studies conducted on both smokeless and smoking tobacco initiation (Sharma et al., 2017; O’Loughlin et al., 2017). Literature also reveals that, earlier the onset of the habit, the greater the dependency on it. (Zickler, 2004). 

Before understanding the factors responsible for tobacco initiation, it is important to understand the existing health beliefs, habits and the upstream factors in the study population. In India smoking of tobacco among women is culturally seen as unacceptable , whereas, the chewing of tobacco has greater acceptability and widespread use. (Chollat-Traquet, 1992; Tomar et al., 2016). This study highlights the greater predisposition of tribal women to chew tobacco, as the men in the community has shifted to smoked forms such as cigarettes and beedis. This calls for gender specific targeted cessation interventions. 

Like smoking, smokeless form of tobacco is addictive and perhaps more difficult to quit (Severson et al., 1985; Henningfield et al., 1997). The addictive nature of tobacco, compounded with the early initiation of the habit, is largely responsible for the profound dependency among the tribes. There is a widespread belief among tobacco chewers across the world, that chewing tobacco is less dangerous than smoking (Chassin et al., 1985; Cohen et al., 1987; O’Connor et al., 2007; Kakde et al., 2012; Chaffe et al., 2019). Present study revealed that tribes were aware of the harmful effects of chewing, but did not believe that it could lead to deadly diseases as such as cancer. The reason stated being they have not seen any examples with such severe outcomes and even if they do, they do not link this to tobacco chewing. 

India is the second largest producer and fourth largest exporter of tobacco with its major share of consumers belonging to the poorer sections of society, which includes the scheduled tribes (Rani et al., 2003; Subramanian et al., 2004; Bhawna, 2013). The flourishing tobacco industry in Wayanad, lack of effective implementation of anti-tobacco laws, ease of accessibility (to even minors) are some of the leading factors for the rampant use of tobacco in this region. This will be a major roadblock when planning tobacco cessation interventions. The ease with which a minor can purchase tobacco or tobacco products could be an important pointer towards the early initiation of tobacco use in this population. (Hoppock and Houston, 1990; Pokorny et al., 2003). 

Ethnography was the chosen study design, as it explores the beliefs and practices of a population. The design allows the research question to be viewed in the context in which they occur and thereby to obtain a deeper understanding of behaviour surrounding health and illness (Savage, 2000). In this study we are looking at the contextual factors contributing to tobacco initiation and how the ethnographic assumptions from the results can contribute to strategies aimed at prevention of habit initiation. Enculturation is the process by which a person adopts the behavioural patterns of the culture he or she lives in (Zimmerman et al., 1998; Les Whitbeck et al., 2004). Tobacco chewing is strongly rooted in the culture of Paniya tribes. It is a part of their customs and festivals and has been practised across generations. Most of the tribes testified that they have grown up seeing their elders chewing tobacco and how normal this habit is perceived in the community. As culture leads to behaviour this factor forms an integral role in habit initiation (Brady, 2000; Mukherjea et al., 2012). 

Tests of social learning theories indicate that parents are strong prospective predictors of habit initiation, this holds true for smoking and tobacco chewing (Akers and Lee, 1996). Majority of the participants said that they started this habit by seeing their parents chew. Even though the current generation of parents restrict their children from chewing, they are not setting an example by refraining from chewing, thereby facilitating initiation. Therefore, the influence of family, particularly parents contributes largely in tobacco initiation compared to other factors, as the first lessons of tobacco smoking and consumption are usually learnt at home (Emmanuel et al., 1991). 

Marriage has an influence on tobacco initiation particularly among women. Many of the tribal women are discouraged in their parental houses to chew tobacco, but after marriage they take up this habit as a sign of adulthood. Their spouse and in-laws also encourage this by providing the tobacco products, thereby initiating the habit. There is also an established association between marital status and higher consumption of tobacco in women. (Ashley and Ferrence, 1998; Rani et al., 2003; Esson et al., 2004; Sinalkar et al., 2015).

The role of peer influence on risk behaviours is a much-researched area and tobacco chewing is no exception to this (Maxwell, 2002). Chewing tobacco is a group activity among tribes and is common when they get together for festivals or community meetings. Testimonials by peer oftentimes, pushes young non-users into experimenting and thereafter falling prey to this habit. The rationale behind this is that adolescents share a common biological environment over a relatively short period of time, these physical changes are coupled with shifting personal expectations and social demands (Miller et al., 2006). Increased availability of tobacco at workplaces coupled with peer pressure is one of the major reasons for initiation of this habit among men.

There is a misconception worldwide across different tribes that tobacco chewing can relieve toothache (Christen et al., 1982). This study reveals, it is the use of tobacco for relieving pain in the gums and teeth, which leads to habit initiation. This signifies the role of dentists in educating and dispelling the myth of tobacco use to treat toothache (Gupta and Ray, 2003). 

When exploring the negative cases who refrain from tobacco chewing, socialization was observed to have played a major role in people to not take up this habit. Paniya tribes being marginalized and less socialized, led to the normalization of this habit among community. Usually people stop chewing when they move out of community and this holds true as peer influence from other sections of society is found to be effective to initiate as well as stop tobacco chewing (Maxwell, 2002). 

The study explored the perceptions of not only key informants who chew, previous chewers and non-chewers were also included in order to get a deeper understanding of the initiation adding to the strength of the study. The well-established participant-researcher relationship has contributed to the quality of the research output as revealing habit initiation and practice of tobacco use involves overcoming social-desirability bias by the participants. Children and adolescent population below 18 years of age, could not be interviewed as our experience with the tribes has been indicative of presence of parental gate keeping. This was one of the drawbacks of the study as getting parental assent was difficult for this population whose perceptions could have contributed to the richness of data. Alternatively, one of the drawbacks include involvement of lesser number of males as key informants which could have affected the transferability of the research work, as substance abuse is more common in males of comparable populations. Adding to that, the gender differences in smokeless tobacco initiation would have been clearer with more male key informants. In-spite of these short comings, the research can be considered as first of its kind to be attempted in the tribal population in question where incidence of oral cancer is on the rise and a deeper understanding of risk factor initiation is warranted for cancer prevention.

In conclusion, the findings from the study stress the influence of socio-cultural factors in smokeless tobacco initiation. The habit starts from adolescence with parental influence and peer pressure playing a major role. The women who are showing a predisposition for chewing in this community are initiated to this habit after marriage. Men being more socialized and employed have shifted to tobacco smoking with a minority continuing smokeless form, initiated by peer pressure and workplace availability. Negative cases revealed the role of socialization and health awareness playing a major role in refraining from this habit. The role of contextual factors like enculturation, marginalization and perceived health benefits also plays a substantial role in habit initiation. This study projects the importance of the control of tobacco initiation in marginalized communities and how important it is to prevent the development of this habit. 

Interventions are suggested aiming at the target groups of adolescents and women of marriageable age. Few strategies which can be applied to similar populations for preventing tobacco initiation based on our findings is given below:

1. De-normalization of this habit by social interaction.

2. Parental awareness on how they influence their children to initiate chewing.

3. Community awareness about the laws of selling tobacco products to minors.

4. Dispelling the myth of tobacco chewing relieving tooth ache by dentists.

5. Educating peer groups and work groups to break the habit together.

6. Creating awareness among young women about the chances of falling into this habit after marriage.
